# Development of Lectin-Linked Immunomagnetic Separation for the Detection of Hepatitis A Virus

**DOI:** 10.3390/v6031037

**Published:** 2014-03-04

**Authors:** Sang-Mu Ko, Joseph Kwon, Bipin Vaidya, Jong Soon Choi, Hee-Min Lee, Myung-Joo Oh, Hyeun-Jong Bae, Se-Young Cho, Kyung-Seo Oh, Duwoon Kim

**Affiliations:** 1 Department of Aqualife Medicine, Chonnam National University, Yeosu, Jeonnam 550-749, Korea; E-Mails: rhtkdan7@naver.com (S.-M.K.); ohmj@jnu.ac.kr (M.-J.O.); 2 Korea Basic Science Institute, Daejeon 305-806, Korea; E-Mails: joseph@kbsi.re.kr (J.K.); jschoi@kbsi.re.kr (J.S.C.); 3 Department of Food Science and Technology and Functional Food Research Center, Chonnam National University, Gwangju 500-757, Korea; E-Mails: bipinvaidya@hotmail.com (B.V.); lhmein@naver.com (H.-M.L.); sycho5633@gmail.com (S.-Y.C.); oks665@naver.com (K.-S.O.); 4 Department of Forest Products and Technology, Chonnam National University, Gwangju 500-757, Korea; E-Mail: baehj@jnu.ac.kr; 5 Bioenergy Research Center, Chonnam National University, Gwangju 500-757, Korea

**Keywords:** hepatitis A virus, soybean agglutinin linked-magnetic bead separation, lectin, oyster

## Abstract

The accuracy and sensitivity of PCR-based methods for detection of hepatitis A virus (HAV) are dependent on the methods used to separate and concentrate the HAV from the infected cells. The pH and ionic strength affect the binding affinity of the virus to cells. In this study, we initially investigated the effects of pH (4.0–10.0) and metal ions (Fe^2+^, Co^2+^, Cu^2+^, Mg^2+^, K^+^, and Ca^2+^) on the binding of HAV to oyster digestive cells. The lowest relative binding (RB) of HAV to the cells was found at pH 4.0 and in FeSO_4_ solution (64.6% and 68.1%, respectively). To develop an alternative to antibody-dependent immunomagnetic separation prior to detection of HAV using RT-PCR, the binding of HAV to five lectins, peanut agglutinin (PNA), *Dolichos biflorus* agglutinin (DBA), *Helix pomatia* agglutinin (HPA), *Ulex europaeus* agglutinin (UEA-1) and soybean agglutinin (SBA), was evaluated using ELISAs. SBA showed significantly higher RB to HAV than the other lectins tested. In addition, HAV could be concentrated within 30 min using SBA-linked magnetic bead separation (SMS) prior to the RT-PCR assay. Our findings demonstrate the feasibility of using SMS combined with RT-PCR to detect HAV at dilutions ranging from 10^−1^–10^−4^ of a HAV stock (titer: 10^4^ TCID_50_/mL).

## 1. Introduction

Viruses are a major cause of foodborne illness, due in large part to the contamination of shellfish by hepatitis A virus (HAV) [1]. HAV, the prototype of the *Hepatovirus* genus within the *Piconaviridae* family, is composed of an icosahedral capsid that contains a positive sense single-stranded RNA genome [2,3]. HAV infection is associated with the consumption of virus-contaminated bivalves, including oysters, clams, cockles and mussels, which accumulate the virus from seawater as they filter-feed [4]. Moreover, the increasingly widespread incidence of oyster-related HAV infection is attracting greater attention to the mechanisms underlying the interaction between HAV and oyster [5].

The detection of HAV is influenced by the binding affinity of the virus for receptor cells, which is affected by the pH and ionic strength of the test solution, the presence of compounds competing for sorption, and the properties of the virus [6]. In general, low pH reduces the infectivity of enteroviruses, as it reduces the stability of virus. However, HAV shows greater stability at low pH than other enteroviruses, which increases the possibility of infection to the cells [7]. Exposing a virus to change in pH can affect binding by altering the conformation of viral proteins e.g., hemagglutinin and spike protein, which respectively mediate the binding of influenza and coronaviruses to the target cells [8,9]. In addition, metal compounds yield specific acids in aqueous solution, so that the ionic strength of a solution will depend on the types of metals present. Consequently, the effects of pH and metal ions on the binding of viruses to cells are considered to be important factors for the detection of viruses in food. To date, however, there have been few studies addressing this issue. Zajac *et al.* reported that Ca^2+^ promotes the interaction between HAV and BS-C-1 cells (monkey kidney cell line) [10], and Bishop *et al.* showed that low pH and divalent metal ions (Mg^2+^, Ca^2+^ and Zn^2+^) enhance the interaction between HAV and BS-C-1 cells due to changes in the viral capsid conformation [11]. Although these studies provide useful information on the binding of HAV to BS-C-1 cells, much remains to be learned about the effects of pH and metal ions on the binding of HAV to different cell types.

Methods investigated as potential approaches for the detection of HAV in oysters include various polymerase chain reaction (PCR) techniques, such as nested-PCR [12,13], reverse transcription (RT)-PCR [14,15,16,17] and real time RT-PCR [18,19]. However, the detection of HAV is not directly compatible with PCR, because the method requires an appropriate concentration of sample, removal of interfering substances and the ability to distinguish between infectious and non-infectious virus particles [20,21]. Although methods for concentrating virus, such as acid adsorption-elution, solvent extraction, polyethylene glycol precipitation and adsorption-elution-precipitation, have been developed to improve the efficiency of viral detection [22,23], these methods are time consuming and are not specific to the target virus. To overcome these limitations, immunomagnetic separation (IMS) has been applied to concentrate viruses present in food or water samples prior to PCR [24,25,26,27,28,29]. With this method, magnetic beads coated with specific antibodies are used to separate specific viral proteins and microorganisms under a magnetic field and extract them from water containing interfering and inhibiting substances. The specificity of antibody-antigen binding ensures detection of the target virus [29,30]. For example, using streptavidin magnetic beads coated with biotinylated human anti-HAV IgG, Lopez-Sabater *et al.* were able to capture HAV from shellfish extract and remove the interfering materials present [30]. Although many investigators have successfully applied IMS prior to RT-PCR to detect viruses, the method currently being used requires expensive antibodies from animal sources. To reduce the cost, an alternative method that replaces the requirement for virus-binding antibodies would be highly desirable.

Lectins, carbohydrate-binding proteins, are ubiquitous in nature [31]. The lectins derived from plant and animal sources are classified based on their ability to bind carbohydrates, such as mannose, galactose, *N*-acetyl-d-galactosamine, *N*-acetylglucosamine, l-fucose and sialic acids [32]. It was previously shown that lectins bind N-acetylglucosamine residues present in the glycoprotein in herpes simplex virus [33]. Moreover, the binding of lectin to HIV and human parainfluenza virus type 2 inhibits the attachment of these viruses to their receptor cells [34,35]. For that reason, many investigators have applied lectins for the separation and isolation of viruses such as herpes simplex [33], Rous-sarcoma [36] and turkey herpes [37], as well as bacteria such as *Streptococcus cricetus* [38], *Bacillus anthracis* [39], enteric bacteria [40] and *Campylobacter* species [41]. Up to now, however, lectin has not been reported for the separation and concentration of HAV.

Our aim in the present study was in part to investigate the effects of pH and metal ions in the binding of HAV to oyster digestive cells. In addition, we investigated the binding of five types of lectin, peanut agglutinin (PNA), *Dolichos biflorus* agglutinin (DBA), *Helix pomatia* agglutinin (HPA), *Ulex europaeus* agglutinin (UEA-1) and soybean agglutinin (SBA), to HAV with the aim of selecting the appropriate lectin for use as an alternative to an antibody in IMS for HAV. Finally, we assessed the feasibility of using the selected lectin in lectin-linked magnetic beads separation (LMS) prior to RT-PCR as a rapid and less expensive method of HAV detection.

## 2. Results and Discussion

### 2.1. Effect of pH and Metal Ions on the Binding of HAV to Oyster Digestive Cells

**Figure 1:**
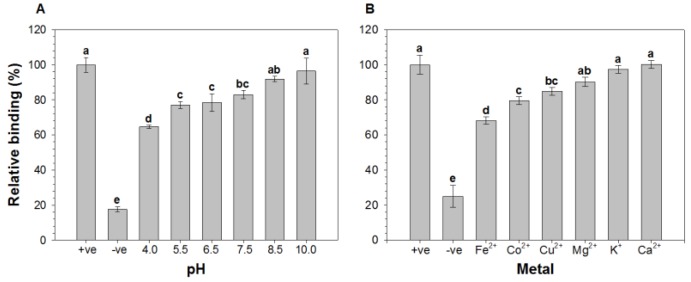
Effect of (**A**) pH and (**B**) metal ions on the relative binding (RB) of hepatitis A virus (HAV) to oyster digestive cells. The cells were exposed to HAV in buffer solutions at the indicated pHs or with the indicated metal ions. The RB was determined using ELISAs by comparing the optical densities of the test samples with that of a positive control (+ve), which consisted of cells exposed to HAV in distilled water. The negative control (−ve) consisted of the cells in distilled water without HAV. Bars labeled with different letters significantly differ from one another (*p* < 0.05; Tukey’s HSD test).

After exposing oyster digestive cells to HAV in buffer solution at selected pHs (e.g., 4.0, 5.5, 6.5, 7.5, 8.5, and 10.0.) or with various metal ions (Fe^2+^ (FeSO_4_), Co^2+^ (CoCl_2_), Cu^2+^ (CuCl_2_), Mg^2+^ (MgSO_4_), K^+^ (KCl), and Ca^2+^ (CaCl_2_)), we used ELISAs to assess the relative binding (RB) of HAV to the cells by comparing the optical densities of test solution to that of positive control, which was prepared by treating the cells with distilled water and HAV (Figure 1). The results showed that the RB of HAV gradually declined as the solution pH decreased from 10.0–4.0 (RB: 96.4% at pH 10 to 64.6% at pH 4 (Figure 1A). Although, the precise reason for the lower RB at acidic pH remains uncertain, it may be associated with a pH-related conformational change within the HAV [42] or the cells. Beside these, the nature of bonding between virus and the cells could be the possible reason for lower binding at acidic pH. As previously described, the binding of viruses to shellfish mucus is either ionic or H^+^ ion bonding, in which, increasing the H^+^ ion concentration, that is, decreasing the pH, weakens the bonding between virus and shellfish mucus [43]. On the contrary, a previous study reported that the binding of the virus particles increased in acidic pH in adsorption/elution extraction method, in which negatively charged membrane was used as binding surface [44]. Sobsey *et al.* also reported that the viruses can be effectively adsorbed to nitro-cellulose and epoxy-fiber-glass filter surfaces from water at acidic pH [45]. The possible reason for difference in binding could be the differences in binding surfaces. On the other hand, Sanchez *et al.* showed that the binding of HAV to erythrocytes was the greatest in acidic medium [46]. They suggested this reflected the role of pH in modulating the interaction between HAV and glycophorin A, its receptor in the erythrocyte cell membrane. This implies that the difference between the RB of HAV to erythrocytes and oyster digestive cells in acidic medium likely reflects a difference in the binding receptors. 

The binding of HAV to oyster digestive cells was also affected by the metals present in the test solution. The RB of HAV to the cells was 68.1, 79.5, 84.9, 90.3, 97.4 and 100.2% in solution containing Fe^2+^, Co^2+^, Cu^2+^, Mg^2+^, K^+^ and Ca^2+^, respectively (Figure 1B). Thus the RB of HAV to the cells was lowest in the solution with Fe^2+^ and highest in solution with Ca^2+^. Previous studies also demonstrated that HAV binding to cell receptors is enhanced by Ca^2+^ ion, which results in decreased binding inhibition of virus from cell receptors [10,47]. Notably, the pH of the Fe^2+^ solution was very close to 4.0, as the pH of the Fe^2+^, Co^2+^, Cu^2+^, Mg^2+^, K^+^ and Ca^2+^ solutions were 3.6, 5.5, 2.8, 6.0, 5.9 and 6.3, respectively. The RB of HAV to the cells in solutions with different pH appears to be nearly comparable with the RB at the corresponding pH produced by different metal ions, though Ca^2+^ is an exception. The possible explanations for the higher RB in the presence of Ca^2+^ are the higher pH (6.3) of the solution and/or a ligand-like action of Ca^2+^ contributing to the formation of the virus-cell complex [10]. Beside these reasons, Ca^2+^ binding may alter the viral capsid structure [11]. In our study, Mg^2+^ showed no significant difference in RB of HAV to the cells. Similarly, in previous study, the variation in concentration of Mg^2+^ did not show a significant difference on HAV binding affinity [10].

### 2.2. Comparison of the Relative Binding of HAV to Lectins

**Figure 2:**
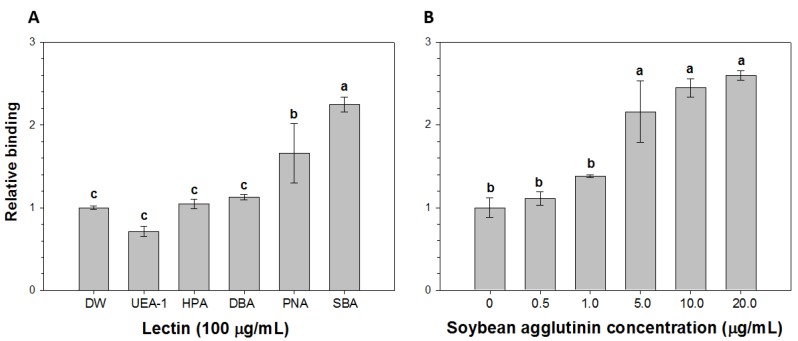
Relative binding (RB) of hepatitis A virus (HAV) to (**A**) peanut agglutinin (PNA), *Dolichos biflorus* agglutinin (DBA), *Helix pomatia* agglutinin (HPA), *Ulex europaeus* agglutinin (UEA)-1 or soybean agglutinin (SBA) and (**B**) SBA at the indicated concentrations. The RB was determined using ELISAs by comparing the optical densities of the test samples (lectins exposed to HAV) with that of a control sample consisting of HAV in distilled water (DW). Bars labeled with different letters significantly differ from one another (*p* < 0.05; Tukey’s HSD test).

The RB of HAV to several lectins (UEA-1, HPA, DBA PNA and SBA) is shown in Figure 2A. The binding was assessed using ELISAs by comparing the optical densities of the test solutions with that of a control solution prepared from distilled water and HAV. The fold changes in the RB of HAV to UEA-1, HPA, DBA, PNA and SBA were 0.7, 1.0, 1.1, 1.7 and 2.3, respectively. Thus, the level of HAV binding was lowest with UEA-1 and highest with SBA. The variation in HAV binding to the different lectins could reflect differences in the carbohydrate specificity of the lectins or specific differences in the interference with HAV binding. The lectins showed different binding specificities such as mannose-, glucose-, galactose-, *N*-acetylglucosamine-, and *N*-acetylgalactosamine-specific binding [48]. For example, SBA preferentially binds galactose/*N*-acetylgalactosamine, whereas UAE-1 preferentially binds l-fucose [32]. Correspondingly, *N*-acetylgalactosamine residue was reported to be recognized by virus for binding indicating that binding of virus to lectin involves specific carbohydrate residue [49].

Given its comparatively high RB of HAV, SBA was selected to bind HAV in LMS prior to RT-PCR assays. Before application of SBA for soybean agglutinin-linked magnetic bead separation (SMS), the concentration-dependent binding of SBA to HAV was investigated by using ELISAs as described above to determine the RB of HAV to selected concentrations of SBA (0–20 μg/mL) (Figure 2B). The fold changes in the RB of HAV to SBA at 0.5, 1.0, 5.0, 10.0 and 20.0 μg/mL were 1.1, 1.4, 2.2, 2.5 and 2.6, respectively. Thus, the RB of HAV to SBA increases with increasing levels of SBA.

### 2.3. Detection of HAV Using SMS Combined with RT-PCR

**Figure 3:**
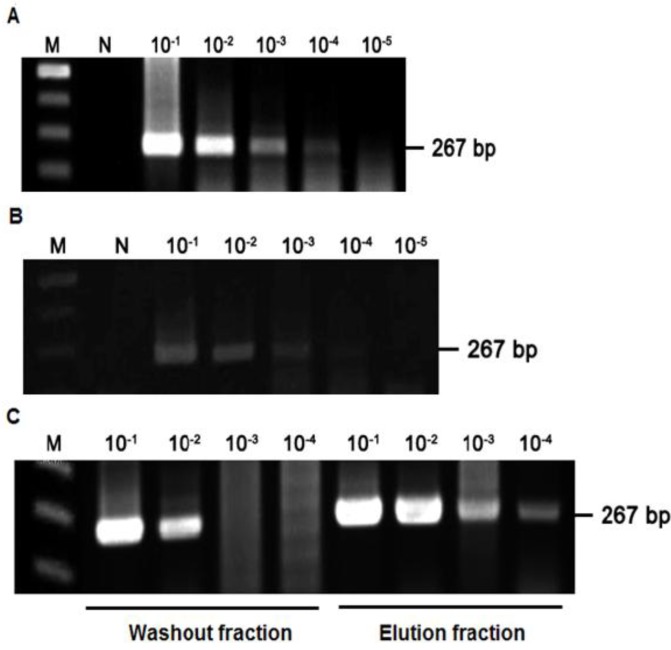
Detection of hepatitis A virus (HAV) using soybean agglutinin-linked magnetic bead separation (SMS) with RT-PCR. (**A**) Determination of the detection limit of HAV using RT-PCR and 10-fold serial dilutions of a HAV stock. M, 100 bp marker; N, negative; (**B**) Determination of the binding of 10-fold serial dilutions (10^−1^–10^−5^) of HAV stock to soybean agglutinin (SBA); (**C**) Detection of HAV using SMS combined with RT-PCR. The limit of detection for the RT-PCR assay was assessed using 10-fold serial dilutions (10^−1^–10^−4^) of HAV (267 bp) present in the washout and eluent fractions. M, 100 bp marker; N, negative.

To determine its limit of detection, we first used RT-PCR to detect the HAV in serial 10-fold (10^−1^–10^−5^) dilutions of the virus (Figure 3A). The amplicon, which was 267 bp, was observed up to a dilution of 10^−4^, indicating the detection limit of RT-PCR assays to be 10^−4^ dilution of our HAV stock (titer: 10^4^ TCID_50_/mL). When we then tested the ability to detect HAV bound to SBA using serial dilutions of HAV treated with SBA (100 µg/mL), the detection limit of the RT-PCR assay was similar to that for HAV without SBA (Figure 3B). Finally, the limit of HAV detection using SMS combined with RT-PCR was measured by preparing serial dilutions (10-fold) of HAV treated with SBA-linked magnetic beads. The HAV that was not bound to the beads was separated as the washout fraction after washing the beads with PBS, while the HAV bound to the beads was separated as the eluent fraction using glycine solvent. The amount and type of eluent such as glycine affect the detection of virus. Therefore, the equal amount of same eluent should be added in all samples to neutralize the effect of eluent in the detection of virus. The HAV in both fractions was detected in RT-PCR assays. Figure 3C shows the amplicons in the washout and eluent fractions were detected at dilutions up to 10^−2^ and 10^−4^, respectively. The detection of HAV in the washout fraction means that the amount of SBA-linked magnetic beads was insufficient for the volume of the sample. These results demonstrate the feasibility of using SMS combined with RT-PCR to detect HAV in food products. However, the method will need to be optimized with respect to the virus concentration and the specificity and quantity of the lectin used. The method could be compatible with the method prescribed by International Organization for Standard (ISO). The organization also prescribed the application of RT-PCR for the detection of targeted viruses including norovirus and HAV in shellfish. In addition, a significant numbers of methods have been reported for the detection of HAV in shellfish; all methods proposed the detection of the virus using RT-PCR with the variation in concentration methods [15,18,50].

## 3. Experimental Section

### 3.1. Oyster Digestive Cells and Virus

Oysters (*Crassostrea gigas*) were purchased from local seafood markets, shucked and dissected to collect the digestive cells. For *in vitro* HAV binding, 1 g of the cells were homogenized in 10 mL of PBS (pH 7.4) using an Omni Tissue Master Homogenizer (Omni International, Kennesaw, GA, USA), after which the homogenates were centrifuged at 3,000 ×*g* at 4 °C for 15 min. HAV strain HM-175/18f (VR-1402) was obtained from the American Type Culture Collection (Rockville, MD, USA). Virus titration was conducted using fetal rhesus monkey kidney cells (FRhK-4) based on the method described by Kim *et al.* [13], and the titer of the HAV stock was 10^4^ TCID_50_/mL.

### 3.2. Relative Binding of HAV to Oyster Digestive Cells Determined by ELISA

ELISAs were conducted to determine the RB of HAV to digestive cells in the presence of selected pHs (4.0, 5.5, 6.5, 7.5 8.5, and 10.0) or metal ions (Fe^2+^ (FeSO_4_), Co^2+^ (CoCl_2_), Cu^2+^ (CuCl_2_), Mg^2+^ (MgSO_4_), K^+^ (KCl) and Ca^2+^ (CaCl_2_)). Fifty µL of diluted oyster digestive cells in distilled water (1:4) were plated in Nunc-Immune plates (96-well ELISA plates, Thermo Fisher Scientific, Roskilde, Denmark) and incubated at 37 °C overnight. The cells were then washed with PBS containing 0.05% Tween 20 (PBS-T), blocked by incubation with 360 µL of 5% skim milk at 37 °C for 1 h. After washing, the cells again with PBS-T, 100 µL of a 1:1 mixture (v/v) of buffer solution with different pHs [prepared by mixing different concentrations of disodium hydrogen phosphate and sodium dihydrogen phosphate (pH range 5.5–10.0) or citric acid (pH 4.0)] or metal ions (10 mM) and HAV (104 TCID50/mL) were added to the plates, and incubated at 37 °C for 2 h. The cells were then washed with PBS-T, and 50 µL of polyclonal anti-HAV antiserum diluted 1:500 in 5% skim milk were added to the plates and incubated at 37 °C for 1 h. The plates were again washed with PBS-T, after which 50 µL of polyclonal rabbit anti-mouse immunoglobulin-HRP (Dako Cytomation, Glostrup, Denmark) diluted in skim milk (1:1000) were added, and the plates were incubated at 37 °C for 1 h. Thereafter, the plates were washed with PBS-T, and 50 µL of 1 mg/mL *o*-phenylenediamine in phosphate citrate buffer (0.2 M sodium phosphate, 0.1 M citric acid) containing 0.01% hydrogen peroxide were added. Then, after treating the plate with 2 N of sulfuric acid to terminate the color development, the optical density at 492 nm was measured using a microplate reader (SpectraMax M2e, Molecular Devices Corp., Sunnyvale, CA, USA). The positive control consisted of digestive cells treated with 100 µL of a 1:1 mixture (v/v) of distilled water and HAV, while the negative control consisted of cells with distilled water prepared under the similar condition. Percent RB was calculated using the following equation: RB (%) = A/B × 100, where A and B were the mean optical densities at 492 nm ± standard deviation of the test sample and the positive control, respectively [4]. 

### 3.3. Relative Binding of HAV to Lectin Determined by ELISA

ELISAs were conducted to determine the RB of HAV to lectins PNA, DBA, HPA, UEA-1 and SBA (Sigma Aldrich, St. Louis, MO, USA). Fifty µL of each lectin in PBS (100 μg/mL) were added to Nunc-Immune plates and incubated at 4 °C overnight. After washing, the plates with PBS, the lectin was blocked by incubation with 360 μL of 5% skim milk at 37 °C for 1 h. The plates were then washed again with PBS-T, and 50 μL of HAV (10^4^ TCID_50_/mL) were added and incubated at 37 °C for 2 h. Thereafter, the relative levels of HAV binding to lectin were determined as described in the previous section.

### 3.4. Soybean Agglutinin Linked-Magnetic Bead Separation (SMS)

SBA was biotinylated using a Biotin-XX Microscale Protein Labeling Kit (Invitrogen, Carlsbad, CA, USA) according to the manufacturer’s instructions. The biotinylated SBA was linked to 200 µg of streptavidin-conjugated magnetic beads (2.8 µm in diameter; Dynabeads M-280 Streptavidin, Invitrogen, Oslo, Norway) and incubated for 30 min at room temperature. The beads were then washed with 200 μL of PBS-T, and samples containing HAV were transferred to the beads and incubated for 30 min at room temperature with rotation at 50 rpm. The beads were then washed again with 200 µL of PBS-T and treated with 0.5 mM glycine (pH 2.8) to elute the virus, after which the eluent was neutralized using 1 M Tris-HCl (pH 9.0).

### 3.5. RT-PCR Assay

Viral RNA was prepared using Trizol-LS reagent (Invitrogen, Carlsbad, CA, USA) with 200 µL aliquots of the HAV-containing washout or eluent fraction. Total cDNA was synthesized from 9.5 µL of the extracted RNA in a reaction mixture that also contained 2 µL of random primer (Takara, Shiga, Japan), 4 µL of 5 × reaction buffer (Beams Biotech., Seongnam, Korea), 2 µL of 50 mM dithiothreitol (Beams), 2 µL of deoxynucleoside triphosphate mixture (Takara, Tokyo, Japan), 0.5 µL of RNase inhibitor (Takara) and 1 µL of Moloney murine leukemia virus reverse transcriptase (200 U/µL; Beams). The reaction mixture was incubated first at 37 °C for 60 min, then at 70 °C for 10 min to inactivate the reverse transcriptase, after which it was incubated at 8 °C for 5 min. PCR to verify the viruses was performed using a pre-mix PCR kit (GeneAll, Seoul, Korea). One µL of cDNA, 1 µL of forward primer (10 pmol/µL), 1 µL of reverse primer (10 pmol/µL), and 17 µL of DNase- and RNase-free water were added to the pre-mix PCR kit. The HAV primer set, VP1_F: 5'-TAT TTG TCT GTC CAC AGA ACA ATC AG-3' and VP1_R: 5'-AGG AGG TGG AAG CAC TTC ATT TGA-3', was used for the PCR analysis [13]. The amplification protocol entailed pre-denaturation at 95 °C for 5 min, followed by 40 cycles of denaturation at 95 °C for 30 s, annealing at 60 °C for 1 min and extension at 72 °C for 1 min, and a final extension at 72 °C for 5 min. The amplified products were separated by electrophoresis on a 1.5% agarose gel and staining with ethidium bromide, after which they were visualized under UV light.

### 3.6. Statistical Analysis

All statistical analyses was carried out using SPSS software (IBM Corp., Somers, NY, USA) [51] and Excel software (Microsoft Corp., Redmond, WA, USA) [52]. All samples were analyzed in triplicate and represent three independent experiments to ensure assay consistency. Analysis of variance was used to compare groups. Significant differences among means were determined using Tukey’s HSD test. Values of *p* < 0.05 were considered significant.

## 4. Conclusions

Our findings indicate the feasibility of using SMS combined with RT-PCR to detect HAV in oyster. By using lectins to bind viruses, the developed SMS eliminates the need for expensive virus-binding antibodies; and when combined with RT-PCR, this method is capable of detecting HAV at levels defined by dilution to 10^−1^–10^−4 ^of a HAV stock (titer, 10^4^ TCID_50_/mL). With further optimization, this approach has the potential for the development of a lateral flow strip test to detect HAV as an alternative to antibodies.
